# Multivariate relationships among nucleus and Golgi properties during fibrillar migration are robust to and unchanged by epithelial-to-mesenchymal transition

**DOI:** 10.1371/journal.pone.0239188

**Published:** 2020-09-18

**Authors:** Catherine Y. Luo, Robert J. Natividad, Mark L. Lalli, Anand R. Asthagiri

**Affiliations:** 1 Department of Bioengineering, Northeastern University, Boston, MA, United States of America; 2 Department of Chemical Engineering, Northeastern University, Boston, MA, United States of America; 3 Department of Biology, Northeastern University, Boston, MA, United States of America; University of Colorado Boulder, UNITED STATES

## Abstract

Epithelial-to-mesenchymal transition (EMT) and maturation of a fibrillar tumor microenvironment play important roles in breast cancer progression. A better understanding of how these events promote cancer cell migration and invasion could help identify new strategies to curb metastasis. The nucleus and Golgi affect migration in a microenvironment-dependent manner. Nucleus size and mechanics influence the ability of a cell to squeeze through confined tumor microenvironments. Golgi positioning determines front-rear polarity necessary for migration. While the roles of individual attributes of nucleus and Golgi in migration are being clarified, how their manifold features are inter-related and work together remains to be understood at a systems level. Here, to elucidate relationships among nucleus and Golgi properties, we quantified twelve morphological and positional properties of these organelles during fibrillar migration of human mammary epithelial cells. Principal component analysis (PCA) reduced the twelve-dimensional space of measured properties to three principal components that capture 75% of the variations in organelle features. Unexpectedly, nucleus and Golgi properties that co-varied in a PCA model built with data from untreated cells were largely similar to co-variations identified using data from TGF*β*-treated cells. Thus, while TGF*β*-mediated EMT significantly alters gene expression and motile phenotype, it did not significantly affect the relationships among nucleus size, aspect ratio and orientation with migration direction and among Golgi size and nucleus-Golgi separation distance. Indeed, in a combined PCA model incorporating data from untreated and TGF*β*-treated cells, scores of individual cells occupy overlapping regions in principal component space, indicating that TGF*β*-mediated EMT does not promote a unique “Golgi-nucleus phenotype” during fibrillar migration. These results suggest that migration along spatially-confined fiber-like tracks employs a conserved nucleus-Golgi arrangement that is independent of EMT state.

## Introduction

Cell extrinsic and intrinsic factors contribute to breast cancer progression. Outside the cell, an increase in the stiffness, density and alignment of collagen fibrils is observed during breast cancer progression [1–4]. Mature collagen fibers provide tracks along which breast cancer cells migrate. Meanwhile, cell-intrinsic epithelial-to-mesenchymal transition (EMT) is associated with cancer progression and metastasis [5, 6]. During EMT, epithelial cells undergo changes in gene expression resulting in loss of cell-cell adhesions and adoption of a migration phenotype. An array of mechanisms, including upregulation of soluble TGF*β*, is implicated in inducing EMT during cancer development [7]. Understanding how EMT affects fibrillar migration will provide insights into the etiology of breast cancer and help identify potential therapeutic targets.

The nucleus and Golgi play key roles in cell migration in a microenvironment-dependent manner. Nucleus deformation is important when cancer cells migrate through pores in a stiff microenvironment. Being the largest organelle, a nucleus with reduced stiffness, as observed in cancer cells, will be more pliable and permit cells to squeeze through confined microenvironments [8–10]. In addition, Golgi positioning relative to the nucleus helps define the front-rear polarity of a migrating cell. In classical 2d tissue culture, the Golgi is often found ahead of the nucleus and may facilitate migration by producing and trafficking adhesion proteins to the leading edge where they mediate traction on the substrate [11, 12]. However, when cells are confined to migrate along narrow fiber-like micropatterns to mimic a fibrillar collagen microenvironment, the Golgi is positioned posterior to the nucleus [13, 14]. We have recently shown that more significant than the position of the Golgi is the stability with which its position is maintained [15]. The stability of Golgi positioning, not whether the Golgi is ahead or behind the nucleus, is associated with EMT-mediated enhancement of fibrillar migration.

As the role of individual features of nucleus and Golgi in migration continue to be clarified, there is a growing need to develop a systems-level understanding of how the manifold features of these organelles work together during migration. Are some features of these organelles inter-related? Are there features of the nucleus associated with certain aspects of the Golgi? Revealing such relationships would provide hypotheses on how certain aspects of the nucleus and Golgi may be functionally connected. Alternatively or in addition, strongly-associated nucleus and Golgi features may suggest a common upstream regulator. A systems-level analysis can also reveal in what ways changes in the microenvironment and/or the intrinsic state of the cell alter the relationships among features of the nucleus and Golgi during cell migration.

To begin to probe systems-level relationships among nucleus and Golgi properties, we quantified and conducted multivariate analysis of twelve morphological and positional properties of these organelles during fibrillar migration. We examined mammary epithelial cells that were either treated with TGF*β* to induce EMT or left untreated to maintain an epithelial state as they migrated along 10 *μ*m fiber-like micropatterns of collagen. We used principal components analysis (PCA) to reduce the twelve-dimensional space of measured properties to three principal components (PCs) that capture 75% of the variations in organelle features. Each PC is an axis through the 12-dimensional space that captures the maximum variation in measured properties. Analyzing these PCs reveals organelle features that exhibit strong co-variations. Most unexpectedly, our analysis shows that a PCA model built with data from untreated cells is remarkably similar to that built from data collected with TGF*β*-treated cells. Despite significant changes in gene expression, morphology and motile phenotype induced by TGF*β*-mediated EMT in these cells [15, 16], treatment with TGF*β* does not significantly affect the relationships among nucleus size, aspect ratio and orientation with migration direction or among Golgi size and nucleus-Golgi separation distance. These results suggest that migration along spatially-confined fiber-like tracks employs a conserved nucleus-Golgi arrangement that is independent of EMT state.

## Materials and methods

### Data set on nucleus and Golgi properties during cell migration

To better understand the complex relationship among nucleus and Golgi properties during fibrillar migration, we modeled and analyzed data gathered in our prior work [15]. Migration of non-transformed human mammary epithelial cells (MCF10A) expressing histone 2B-GFP (nuclear marker) and GM130-RFP (Golgi marker) was imaged on micropatterned lines of collagen as detailed in [15]. MCF10A cells were maintained and passaged using standard growth medium and tissue culture protocols [16, 17]. Additionally, to analyze how EMT affects relationships among nucleus and Golgi properties, MCF10A cells were treated with 20 ng/mL TGF*β* in growth medium for 12 days. Transformation to mesenchymal state was confirmed through analysis of morphological (cell shape factor, loss of cell-cell contacts) and protein expression (E-cadherin and N-cadherin) as described in previous work [16]. TGF*β*-treated and untreated MCF10A cells were seeded onto micropatterned 10 *μ*m wide collagen tracks to model migration in the fibrillar TME. Collagen tracks were produced through micro-contact printing as described in past work [15, 18]. Migration along these pseudo-1D tracks was recorded with time-lapse confocal microscopy with images acquired every 2.5 min.

Automated segmentation of time-lapse images of fluorescently labeled migrating cells was completed in MATLAB using a custom program described in prior work [15]. Briefly, the regionprops function was used to calculate the area, perimeter, and centroid of the nucleus and Golgi. All other nucleus and Golgi parameters were calculated from these values.

### Data preprocessing

To focus analysis on the variations in nucleus and Golgi properties without bias from different scales of magnitude in their values, the measured values of properties were mean-centered and standard deviation-normalized in MATLAB prior to input into the PCA algorithm using

$$\begin{array}{c} p_{ij}=\frac{p_{ij}^{*}-\bar{p_{i}}}{\sigma_{p_{i}}} \end{array}$$ (1)

where $p_{ij}^{*}$ and *p*_*ij*_ are the values of property *i* for cell *j* before and after scaling, respectively, and $\bar{p_{i}}$ and $\sigma_{p_{i}}$ are the mean and standard deviation of property *i* across all cells *j* = 1…*N*. For untreated cells *N* = 49, and for TGF*β*-treated cells *N* = 61. The total number of properties measured for each cell is *P* = 12.

### Principal component analysis

PCA was performed to model the data set of nucleus-Golgi properties (*P* × *N* matrix ***P***) onto a reduced *K*-dimensional principal space with minimal square error [19]. The orthogonal axes of the new principal component space are given by the column vectors of the *P* × *K* loading matrix ***L***, and the position of each cell in PC space is given by the column vectors of the *K* × *N* scores matrix ***S***. The PCA model is given by

$$\begin{array}{c} P=LS+E \end{array}$$ (2)

where ***P*** contains mean-centered values and ***E*** is the residual.

#### Eigenvalue decomposition

The columns of the loadings matrix are the eigenvectors of the covariance matrix (***PP***^*T*^) of the data set. The eigenvalues are proportional to the amount of variance captured by the associated principal component. Thus, eigenvectors are arranged by descending eigenvalues, with the first column of the loadings matrix representing the first principal component.

PCA by eigenvalue decomposition was performed using the pca function in MATLAB. Since eigenvalue decomposition does not accommodate missing data, preliminary models were built with pairwise deletion by calling pca function with parameters Algorithm to eig and Rows to pairwise.

#### Altenating least squares (ALS)

ALS is an effective algorithm for PCA when the data matrix has few missing data entries [20]. To avoid overfitting, the number of principal components *K* is chosen to be low relative to number of properties (*P*) in the data set. With *K* specified at the outset, ALS was used to find ***L*** and ***S*** in an iterative manner. Starting with matrices seeded with random values, the scores matrix ***S*** was calculated for a given loadings matrix ***L*** using

$$\begin{array}{c} S={\left(L^{T}L\right)}^{-1}L^{T}P, \end{array}$$ (3)

and then a new loadings matrix was calculated from ***S*** using

$$\begin{array}{c} L=PS^{T}{\left(SS^{T}\right)}^{-1}. \end{array}$$ (4)

This alternating process was repeated until reaching convergence.

ALS was performed in MATLAB by calling the pca function with parameters Algorithm set to als and NumComponents set to 3. A random, normally distributed matrix was used as the initial guess for loadings and scores, which was then repeatedly updated until convergence to the final model was reached. Convergence is reached when either the cost-function or the relative change in elements of the loading and/or scores matrices are below the termination tolerance of 10^−6^.

The outputs of the pca function, the loadings and scores matrices and PC variance vector, were used for subsequent analysis. MATLAB was used to generate all plots and perform pairwise comparisons of scores.

### Confidence intervals by bootstrap

The variance accounted for (VAF) by each principal component (PC) and the loadings that define each PC are outputs of the PCA model. To determine the confidence intervals in the estimated VAF and loadings, we employed a bootstrap approach [21, 22] wherein the data set of nucleus-Golgi properties were resampled with replacement to generate *B* = 1000 bootstrap sample sets. PCA was performed and VAF_b*_ and loading matrix $L_{b}^{*}$ were determined for *b* = 1…*B* (asterisk denotes values for bootstrapped sample sets). Procrustes transformation was performed to orthogonally rotate and reflect $L_{b}^{*}$ toward the loading matrix (***L***) derived for the original data set, as given by

$$\begin{array}{c} L=\mu_{b}L_{b}^{*}T_{b}+\beta_{b} \end{array}$$ (5)

where ***T***_***b***_, *μ*_*b*_ and ***β***_***b***_ are the transformations needed to rotate/reflect, rescale and translate, respectively. Only ***T***_***b***_ was used for orthogonal rotation and reflection, with no rescaling and translation. With VAF_b*_ and $L_{b}^{*}T_{b}$, 95% confidence intervals were determined in MATLAB by bias-adjusted, accelerated approach [21–23] using bootci function with bca parameter.

## Results

### Quantification of nucleus and Golgi properties in migrating cells

We quantified eight morphological features of the Golgi and nucleus which have potentially important roles in cell migration (Table 1, properties 5-12). These features were measured from time-lapse images acquired in prior work [15]. Fluorescently-labeled Golgi and nucleus were imaged in non-transformed MCF-10A human mammary epithelial cells migrating on fiber-like micropatterns. Additional information on experimental procedures are provided in the subsection *Data set on nucleus and Golgi properties during cell migration* in Materials and Methods, and a sample video of recorded nucleus and Golgi motion during cell migration can be found in our prior work [15].

**Table 1:** Twelve morphometrics of nucleus and Golgi during fibrillar migration.

Label	Property	Unt c.v.	TGF*β* c.v.
1	Fraction time spent with GPRN Ahead*	0.45	0.90
2	Fraction time spent with GPRN Behind*	0.54	0.47
3	Stability of GPRN Ahead*	0.83	1.2
4	Stability of GPRN Behind*	1.2	1.6
5	Nucleus-Golgi (NG) separation	0.60	0.53
6	Golgi area	0.56	0.83
7	Golgi perimeter	0.56	0.75
8	Nucleus area	0.47	0.62
9	Nucleus perimeter	0.34	0.39
10	Nucleus deformation	0.20	0.20
11	Nucleus aspect ratio	0.22	0.32
12	Nucleus orientation	0.83	0.95

Golgi and nucleus properties were quantified for each cell migrating on fiber-like micropatterns. The coefficients of variation (c.v.) for each property was determined for untreated (Unt) cells (*n* = 49 from 3 independent trials) and TGF*β*-treated cells (*n* = 61 from 6 independent trials). The labels (1-12) are used to identify properties in subsequent charts. Nucleus orientation (12) refers to nucleus orientation with respect to direction of migration. Asterisk denotes properties that were calculated and reported in [15].

In the present work, custom MATLAB code was used to segment the nucleus and Golgi and quantify their area, perimeter, and centroid. At each time point, the area and perimeter of the regions in the image corresponding to the nucleus and Golgi were quantified using the MATLAB regionprops function. Unlike a single contiguous shape that defines the nucleus, the Golgi is a multi-body complex. The area and perimeter of the Golgi complex was calculated as the sum of these properties of all Golgi bodies. The centroid of the Golgi complex was computed as the area-weighted average centroid of each Golgi particle.

Additional nucleus parameters were calculated to assess the role of nucleus deformation in fibrillar migration. Nucleus deformation was calculated as the ratio of nucleus perimeter to nucleus area, with a greater value indicating further deviation from a perfect circle. The MATLAB regionprops function was used to determine the major and minor axis lengths of the ellipse from the second-order central moments of the region. Nucleus aspect ratio was calculated as the ratio of the major to minor axis lengths.

To gauge how the nucleus was situated in the context of the cell body, nucleus orientation was calculated as obtained using regionprops to compute the angle of the major axis of the nucleus with respect to the direction of migration. Additionally, nucleus-Golgi (NG) separation was calculated as the distance between the centroids of the nucleus and Golgi complex at each time point.

To these morphological properties, we added four positional measurements from our prior work (Table 1, properties 1-4). Our group investigated the role of Golgi positioning relative to the nucleus (GPRN) in the enhanced fibrillar migration of cells that have undergone EMT [15]. Centroids of Golgi and nucleus were used to score GPRN at every time-step of cell movement. GPRN state was identified as Ahead or Behind if the Golgi was anterior or posterior, respectively, to the nucleus in the direction of migration. A third Unknown state was assigned when the Golgi or nucleus was not observable or when the cell did not move. From the time-course of observation for each cell, the fraction time the cell spends with GPRN in each of these states was determined. In comparison to Ahead and Behind states, the Unknown state was shown to occur highly infrequently and was therefore negligible. The stability of each GPRN was also quantified based on the duration of time cells contiguously maintained a particular state. Altogether, four metrics of GPRN (fraction time in Ahead/Behind states and stability of Ahead/Behind states) are utilized in the present work.

For each cell, the average of these nucleus and Golgi properties were computed over the full duration of observation. The eight new and four previously-calculated morphometrics are summarized in Table 1 and provide a unique single-cell data set of the state of the Golgi and nucleus during fibrillar migration. The coefficients of variation for these properties show significant variability at the single-cell level within untreated and TGF*β*-treated populations. We asked whether closer examination of this single-cell variability might reveal underlying correlations among a subset of organelle properties.

### Handling missing values when developing multivariate models of Golgi-nucleus properties in untreated versus TGF*β*-treated cells

To what extent nucleus and Golgi are co-regulated during fibrillar migration is poorly understood. We reasoned that co-regulated properties would exhibit high correlation. To test this hypothesis, we performed PCA to identify significant co-variations among nucleus and Golgi properties in cells migrating along 10 *μ*m fiber-like micropatterns.

PCA yields an ordered set of principal components (PCs), each one being a linear combination of measured properties. The first PC is a grouping of measured properties that captures the greatest amount of variation in the data set. Successive PCs capture progressively less variation. The loadings of each Golgi and nucleus property within individual PCs provide insights into the relative strengths of co-linear variations among the properties.

We hypothesized that since EMT causes major changes in gene expression and motile phenotype, migrating cells that have undergone transition to a mesenchymal phenotype may exhibit distinct relationships among Golgi and nucleus properties compared to their non-transformed counterparts. To test this hypothesis, we built separate PCA models of untreated and TGF*β*-treated cells with the goal of comparing the models. We previously showed that MCF-10A mammary epithelial cells treated with TGF*β* undergo changes in gene expression, cell morphology and cell-cell adhesion consistent with EMT [16].

To construct PCA models, we developed a strategy to deal with missing values in the data set. For some individual cells, measurements of GPRN stability are indeterminable. When a cell occupies only one GRPN state through its entire observation time, the other GPRN state never occurs and its stability is physically undefined. Thus, missing values are not due to technical challenges with measurement but because these values are physically undefined for those particular cells. Missing values occur in 4 out of 49 untreated cells and in 8 of the 61 TGF*β*-treated cells.

Several methods have been developed for performing PCA with missing values [24]. One method involves listwise deletion to remove observations (i.e., cells) containing a missing value. However, deletion risks loss of valuable information, as cells that only occupy one GPRN state for the duration of observation may exhibit distinct nucleus and Golgi organization. A second approach is eigenvalue decomposition of the covariance matrix constructed using pairwise deletion. This method does not return score values for observations containing missing data, also resulting in a loss of information. Other methods have been described which involve imputing missing values prior to PCA. In the context of our data, imputation prior to PCA assigns values to GPRN stability that are physically undefined, potentially creating artificial trends in the data.

Here, we use a method known as Alternating Least Squares (ALS) which repeatedly factorizes the data matrix until convergence is achieved to form the PC space [25]. The number of dimensions of the PC space is an input parameter. The ALS algorithm effectively ignores missing data while performing PCA. After the model is developed, missing values can be estimated by reconstructing the data from the PCA scores and loadings. An advantage of ALS over pairwise deletion is its ability to produce full score and loading matrices for all variables and observations, allowing for complete interpretation of how observed cells behave in the PC space. MATLAB was used to implement the ALS algorithm using mean-centered and standard deviation-normalized data.

**Fig 1:**
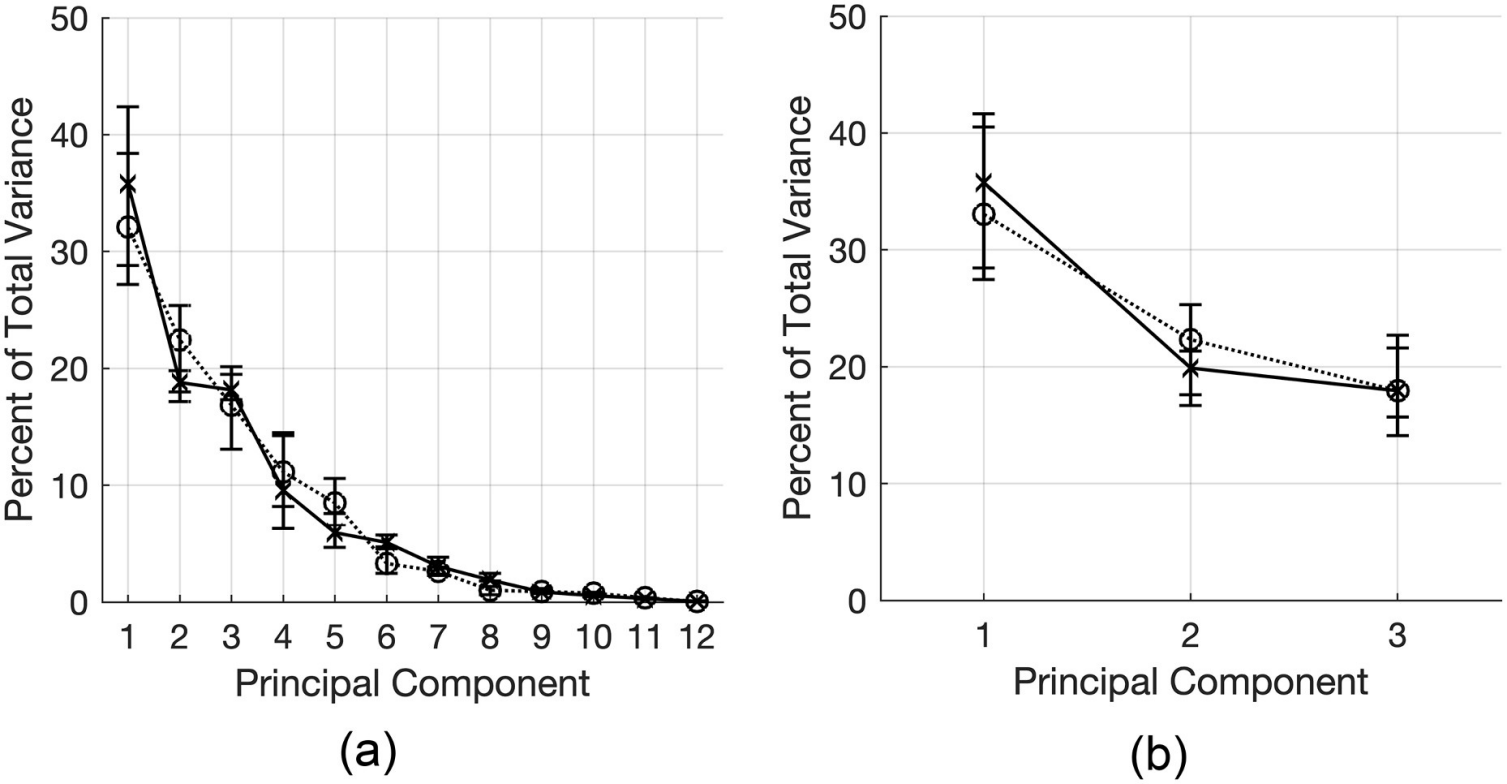
Variance in data captured by principal components (PCs). A: PCA model derived by eigenvalue decomposition following pairwise deletion. The percent of variance in the data set accounted for by each PC is plotted for untreated (solid, x; *n* = 49) and TGF*β*-treated (dotted, circle; *n* = 61) cells. Earliest PCs explain the greatest percentage of total variance in the data set. A 50% drop in the amount of variation captured is observed from PC3 to PC4. B: Three-component PCA model constructed by ALS for untreated cells (solid, x; *n* = 49 from 3 independent trials) and TGF*β*-treated cells (dotted, circle; *n* = 61 from 6 independent trials). Error bars denote 95% confidence intervals determined by bootstrap analysis and bias-adjusted, accelerated method.

To avoid overfitting and achieve reproducible models through ALS, we first determined the minimum number of PCs needed to capture most of the variation in the data set. A model was first generated using eigenvalue decomposition after pairwise deletion of missing values. Based on the eigenvalues (variance) associated with the PCs computed by eigenvalue decomposition, we determined that approximately 70% of the total variance in the data set is captured by the first three components of the PCA model (Fig 1A). Whereas PC2 and PC3 each capture approximately 18–19% variance in untreated data and 17–22% variance in TGF*β*-treated data, PC4 captures 9.5% and 11% of the variance in untreated and TGF*β*-treated cells, respectively. Thus, we identified three components as sufficient for a minimal model that captures most of the variation in the data.

ALS was performed using the loadings from eigenvalue decomposition as initial guesses for a 3-component model. Repeating ALS using random normally-distributed values as the initial guess produced the same loadings and scores, indicating convergence to a consistent model. We then proceeded with analysis using the loadings and scores of the model computed by ALS with a random initial guess.

In both untreated and TGF*β*-treated models developed by ALS, about 73% of the total variance in the original data set is captured by the first three PCs (Fig 1B). The variance captured by each component is comparable in both treatment groups. We next analyzed and compared the loadings of Golgi and nucleus properties along each PC between the untreated and TGF*β*-treated cells.

### Nucleus morphological properties exhibit relationships that are preserved following TGF*β*-mediated EMT

**Fig 2:**
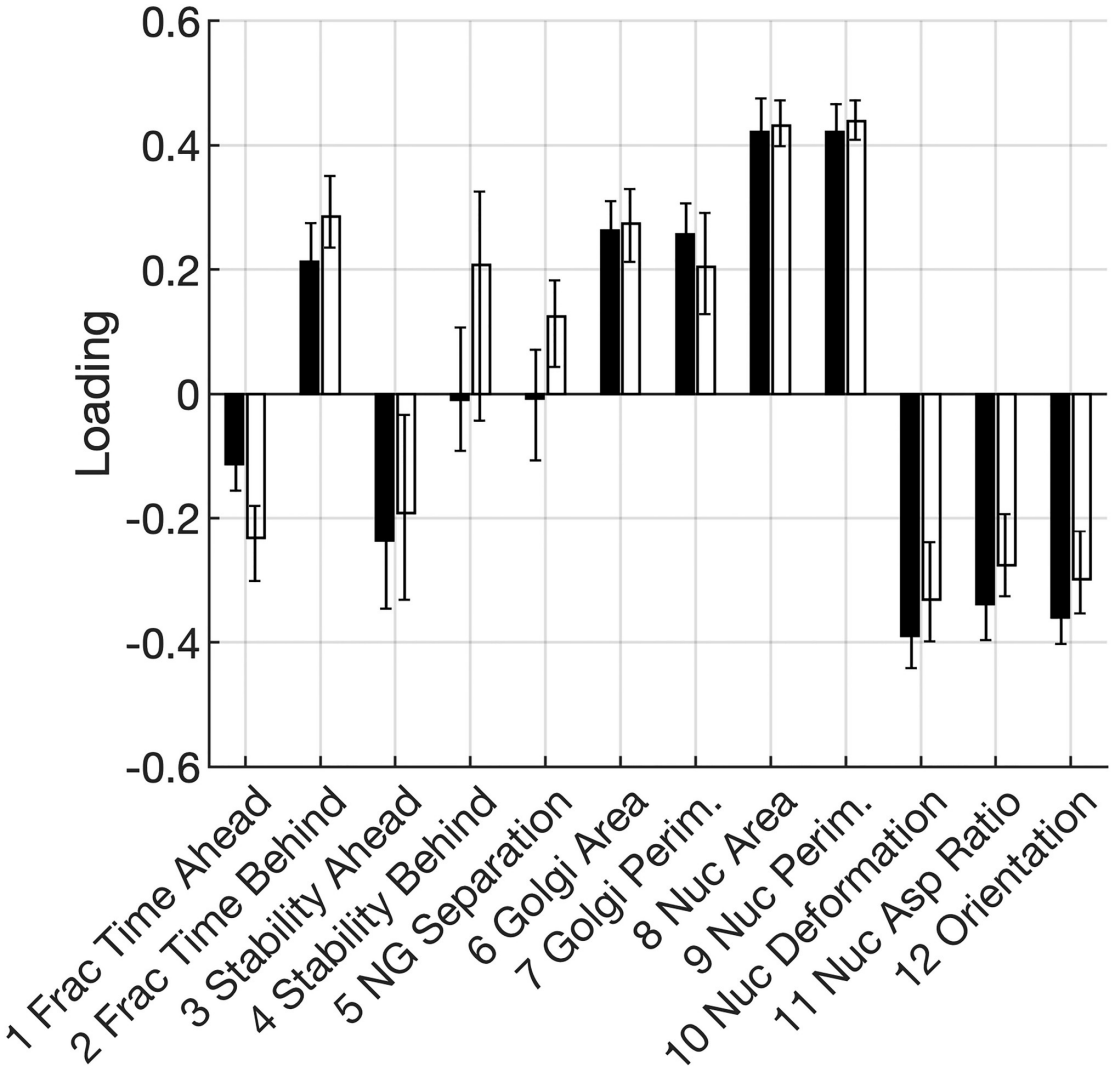
Loadings on the first PC. PC1 loadings on nucleus and Golgi properties for untreated (black; *n* = 49 from 3 independent trials) and TGF*β*-treated (white; *n* = 61 from 6 independent trials) cells. Error bars denote 95% confidence intervals determined by bootstrap analysis and bias-adjusted, accelerated method.

The first PC is by and large identical in PCA models constructed from data for untreated cells versus TGF*β*-treated cells (Fig 2). The loadings for nearly every Golgi and nucleus property have similar magnitude and same sign between the two models. Thus, the co-variations identified by the first PC are Golgi-nucleus relationships that are preserved even after TGF*β*-mediated EMT.

The strongest relationships in the first PC are among nucleus morphological properties (properties 8-12), which have the highest loadings in both models. Nucleus area and perimeter are positively correlated and exhibit a negative correlation with deformation. These geometric relationships are consistent with a typical elliptical geometry of a 2d projection of a nucleus.

Unexpectedly, PC1 shows that nucleus aspect ratio and its orientation with respect to the direction of migration exhibit negative correlation to nucleus size (area, perimeter). Thus, smaller nuclei tend to have a larger aspect ratio and orient orthogonal to the direction of migration, both in untreated and TGF*β*-treated cells.

Relative to nucleus properties, Golgi properties have lower absolute loadings on PC1. Although co-variations among the Golgi properties are not as large as those among the nucleus features, the relationships among Golgi properties are preserved for the most part between untreated and TGF*β*-treated cells. The two exceptions are moderately-loaded (stability behind, 4) and weakly-loaded (nucleus-Golgi separation, 5) in the TGF*β*-treated model and are unloaded in the model of untreated cells.

In summary, the first PC reveals that the largest fraction of total variation in Golgi and nucleus properties (approx. 35%) involve relationships that are common to epithelial cells and counterparts that have undergone TGF*β*-mediated EMT.

### Second and third principal components show relationships among Golgi properties that are common to untreated and TGF*β*-treated cells

**Fig 3:**
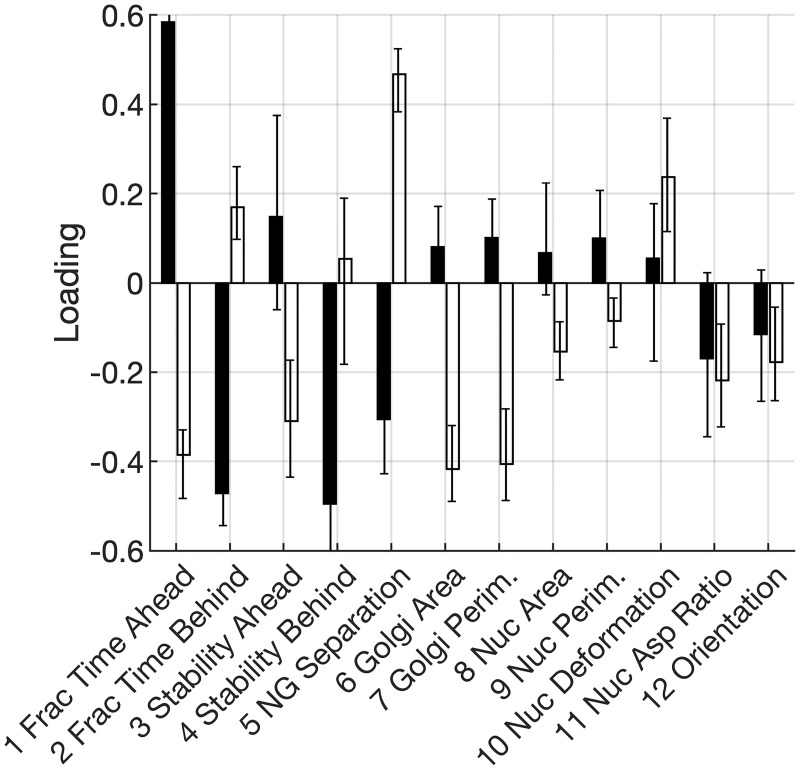
Loadings on the second PC. PC2 loadings on nucleus and Golgi properties for untreated (black; *n* = 49 from 3 independent trials) and TGF*β*-treated (white; *n* = 61 from 6 independent trials) cells. Error bars denote 95% confidence intervals determined by bootstrap analysis and bias-adjusted, accelerated method.

**Fig 4:**
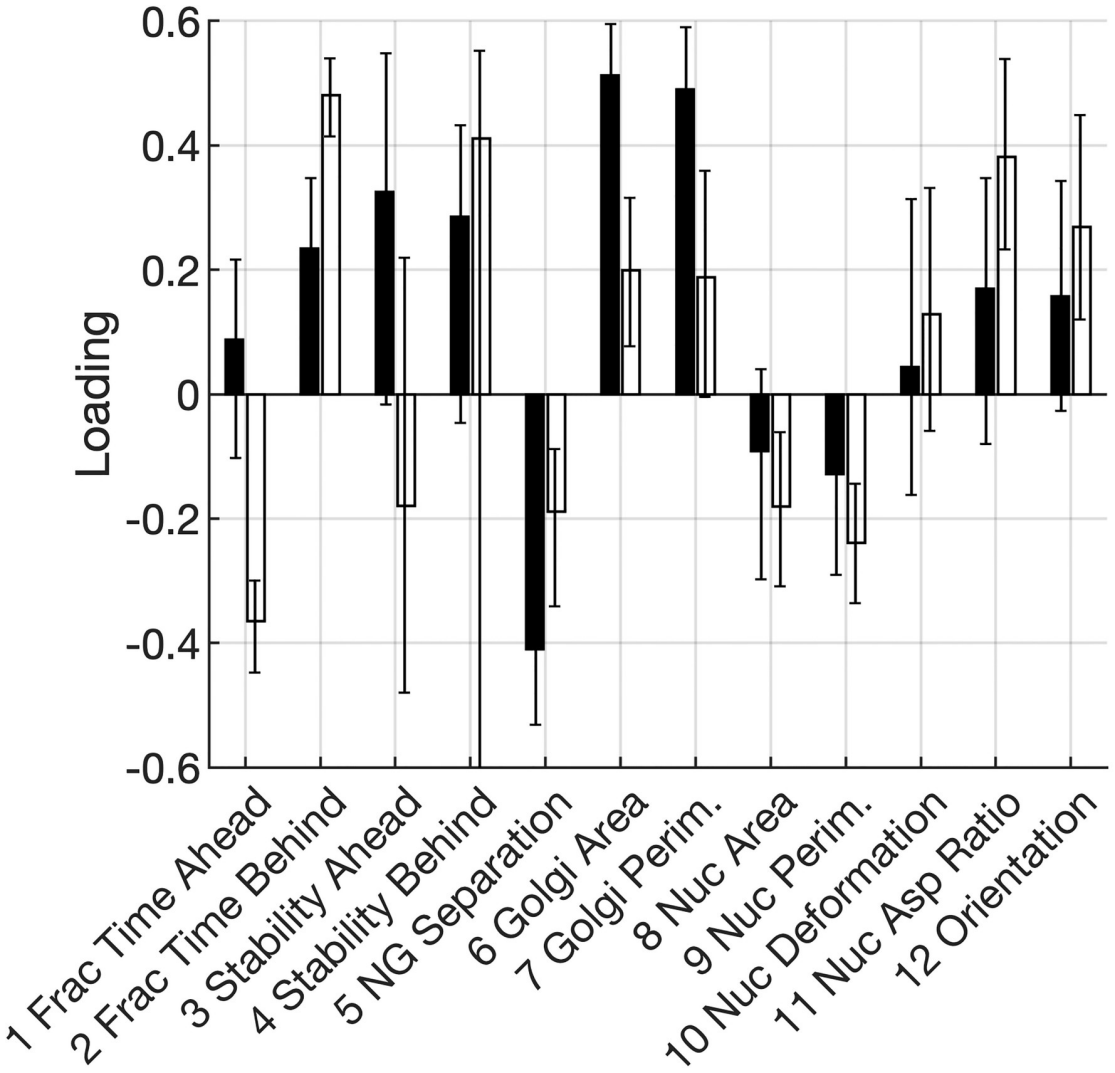
Loadings on the third PC. PC3 loadings on nucleus and Golgi properties for untreated (black; *n* = 49 from 3 independent trials) and TGF*β*-treated (white; *n* = 61 from 6 independent trials) cells. Error bars denote 95% confidence intervals determined by bootstrap analysis and bias-adjusted, accelerated method.

Next, we analyzed loadings along PC2 and PC3. Whereas nucleus morphology makes the largest contribution to PC1, Golgi morphology and Golgi-nucleus positional properties are the dominant factors in PC2 and PC3 (Figs 3 and 4). With the exception of nucleus aspect ratio in PC3 of the TGF*β*-treated model, nucleus morphological properties are weakly-loaded on PC2 and PC3, with loading values at or below approximately 0.2. In contrast, several Golgi-related properties have loadings that approach or exceed 0.4.

Unlike PC1, loadings along PC2 are different in the models for untreated and TGF*β*-treated cells. Golgi area and perimeter are significantly correlated along PC2 in TGF*β*-treated cells but make almost no contribution to PC2 among untreated cells. Meanwhile, Golgi-behind properties are significantly loaded in PC2 for untreated cells but not among TGF*β*-treated cells.

Differences between untreated and TGF*β*-treated cells are also evident along PC3. Golgi area and perimeter exhibit high loadings on PC3 in the model of untreated cells but not in the model of TGF*β*-treated cells. Golgi-behind state properties make only moderate contributions to PC3 among untreated cells but contribute strongly to PC3 in TGF*β*-treated cells.

While PC2 and PC3 individually exhibit differences between untreated and TGF*β*-treated cells, we note that these PCs are nearly equivalent in terms of the fraction of variation that each captures. Thus, if a correlation observed along one component (e.g., PC2 of model with untreated cells) occurs with similar magnitude along the other component (e.g., PC3 of model with TGF*β*-treated cells), it is reasonable to conclude that this correlation is equally significant in both untreated and TGF*β*-treated cells.

With this rationale, we cross-compared PC2 and PC3 between the two models. We find, for example, that Golgi positional properties are highly loaded on PC2 of untreated cells and on both PC2/3 in models of TGF*β*-treated cells. Along these equivalent PCs in both models, Golgi-behind properties are consistently inversely correlated with Golgi-ahead properties. Thus, the inverse relationship between Golgi-ahead and Golgi-behind properties is independent of TGF*β* treatment.

Similarly and more unexpectedly, Golgi size (area and perimeter) and nucleus-Golgi separation distance have high loadings with equal magnitude but opposite signs on PC3 for untreated cells and PC2 for TGF*β*-treated cells. Thus, independent of TGF*β* treatment, large Golgi tends to be positioned closer to the nucleus.

In this manner, cross-comparisons of loadings between PC2 and PC3 reveal that untreated and TGF*β*-treated cells exhibit highly similar relationships among Golgi properties. Taken together with the high similarity in loadings on PC1, our analysis indicates that untreated and TGF*β*-treated cells exhibit a common set of co-variations in nucleus and Golgi properties during fibrillar migration.

### A combined model of variations in Golgi and nucleus properties does not distinguish individual untreated and TGF*β*-treated cells

Analysis of loadings demonstrated that the most significant relationships among nucleus and Golgi properties are similar between untreated and TGF*β*-treated cells. To investigate this further, we developed a combined PCA model with data from both treatment conditions. We asked whether within the PC space of the combined model, untreated and TGF*β*-treated cells would exhibit distinct scores. If untreated and TGF*β*-treated cells show similar scores along PCs in the combined model, the co-varying properties captured by the model are inadequate to distinguish whether a cell has undergone TGF*β*-induced EMT. Alternatively, untreated cells may exhibit scores that are different from counterparts that are treated with TGF*β*, leading to untreated and TGF*β*-treated cells clustering into different subregions of the PC space of a combined model. If such a segregation in PC space is observed, it would demonstrate that co-varying properties of the Golgi and nucleus change in response to TGF*β* treatment and could be used to distinguish untreated epithelial cells from TGF*β*-treated cells that have undergone EMT.

**Fig 5:**
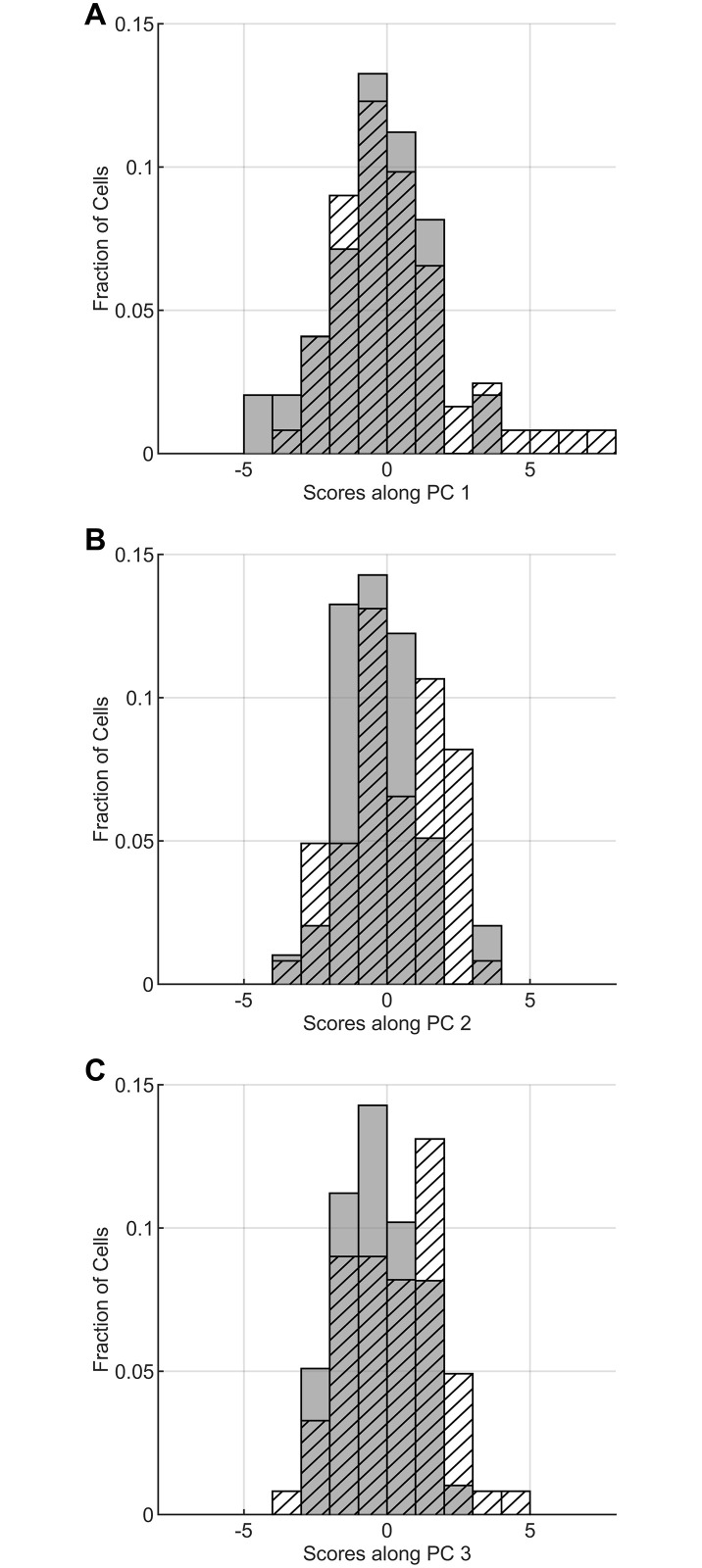
Scores from a combined PCA model of untreated and TGF*β*-treated cells. Scores for untreated (shaded grey; *n* = 49 from 3 independent trials) and TGF*β*-treated (hatched; *n* = 61 from 6 independent trials) cells along A: PC1, B: PC2, C: PC3.

To conduct this test, we generated a PCA model with the combined data from untreated and TGF*β*-treated cells and asked to what extent TGF*β* treatment promotes a distinguishable “Golgi-nucleus phenotype” in PC space. We computed the scores for each cell along the three PCs of the combined model. Along each PC, the distribution of scores for TGF*β*-treated cells is right-shifted relative to that for untreated cells (Fig 5). The mean scores for each cell population are shown in Table 2. Mean values for the TGF*β*-treated population are higher than that for untreated cells on each PC, and pairwise comparisons of untreated and TGF*β*-treated scores are significant for PC2 and PC3. Thus, TGF*β*-induced EMT promotes a distinguishable population-level effect in PC space. However, there is considerable overlap in the distribution of scores among untreated and TGF*β*-treated cells. Thus, the score associated with any individual cell is insufficient to predict treatment with TGF*β*.

**Table 2:** Population-averaged scores.

PC	Treatment	Mean	S.D.	p-value
1	Unt.	-0.3413	1.7258	0.0511
	TGF*β*	0.2742	2.1914	
2	Unt.	-0.3037	1.2629	0.0297
	TGF*β*	0.2439	1.7468	
3	Unt.	-0.3074	1.1616	0.0195
	TGF*β*	0.2469	1.6163	

Mean and standard deviation of scores along each PC were computed for untreated cells and for TGF*β*-treated cells. p-values are calculated for pairwise comparison, testing the alternative hypothesis that the population mean of TGF*β*-treated cells is higher than that of untreated cells.

## Discussion

The most unexpected finding from our multivariate analysis is that TGF*β*-mediated EMT has little effect on the relationships among twelve morphological and positional properties of the Golgi and nucleus in mammary epithelial cells migrating on fiber-like micropatterns. In a PCA model derived from data on untreated cells, the co-variations revealed by the loadings of nucleus and Golgi properties are highly similar to those found in a separate PCA model constructed with data from TGF*β*-treated cells. Corroborating the similarity, a single PCA model that combines data from untreated and TGF*β*-treated cells shows that the scores of individual cells lie approximately in the same region of a three-dimensional PC space regardless of whether these cells were treated with TGF*β*.

Since EMT causes profound changes in gene expression and migration phenotype [5, 6], it is surprising that the underlying co-variations among nucleus and Golgi properties are similar between untreated and TGF*β*-treated cells. This finding raises at least two major questions. What were some of the interesting relationships among Golgi and nucleus properties, and why might relationships among nucleus and Golgi properties be so similar between untreated and TGF*β*-treated cells?

### Relationships among Golgi and nucleus properties

Nucleus properties—area, perimeter, deformation, aspect ratio and orientation with migration—are highly inter-related independent of TGF*β* treatment, as they were the most heavily loaded variables of the first principal component (PC1). One of the relationships has potential relevance to the role of the microenvironment on the configuration of the nucleus. The loadings on PC1 show that smaller nuclei tend to be elongated (higher aspect ratio) and orient orthogonal to the direction of migration. An orthogonal orientation may be permissible to migration on fiber-like micropatterns printed on a 2d surface; however, a different relationship between elongated nuclei and orientation may be necessary to enable effective migration through narrow channels in a 3d matrix where the nucleus can be a physical impediment [8–10].

Golgi properties, meanwhile, were the dominantly-loaded variables on the second and third principal components. Notably, these two components together capture approximately 40% of variation in the entire data set, a level on par with 35% of variation explained by PC1. An unexpected relationship uncovered by the loadings on PC2 and PC3 was that smaller Golgi (area, perimeter) tend to be further from the nucleus in both untreated and TGF*β*-treated cells.

### Potential reasons why relationships among Golgi and nucleus are independent of TGF*β*

Several factors could play a role. First, the fiber-like microenvironment may constrain the Golgi and nucleus to work together in a microenvironment-specific manner to achieve or permit migration. We and others have shown that different cell types migrating on narrow tracks acquire a common uniaxial, extended morphology [14, 18]. Thus, extracellular spatial constraints impose a common shape on the cell body that, in turn, may lead to a conserved Golgi-nucleus arrangement during fibrillar migration.

A second possibility deals with the cell-intrinsic state. In this work, the migration of isolated cells is studied. Typically, epithelial cells are in contact with neighbors. The absence of cell-cell contact may itself trigger a partial EMT-like program in isolated untreated cells. Indeed, in cultures of epithelial clusters, cells at the edge of a cluster have a free edge exposed to the substrate and express mesenchymal markers [26]. Furthermore, heterogeneity at the single-cell level could play a role: not all TGF*β*-treated cells undergo the same extent of EMT. Morphological analysis of TGF*β*-treated MCF-10A cells shows that cell shape factor (Perimeter^2^/4*π* Area) exhibits considerable variability, with a c.v. of 32% [16]. At the level of cell behavior, a fraction of TGF*β*-treated cells exhibit enhanced fibrillar migration, while others migrate with speed and persistence similar to untreated cells [15]. Heterogeneous response to TGF*β* is also observed *in vivo*: a subset of squamous cell carcinoma stem cells reduce cell cycle activity in response to TGF*β*, whereas non-responsive neighbors continue to proliferate at a higher rate [27]. A potential source of this heterogeneity is the complexity of EMT itself. Instead of a two-state model, EMT is better represented as a spectrum, including hybrid or partial E/M states [28, 29]. Relating TGF*β*-induced continuum of EMT states to organelle organization is a subject for future investigation. Meanwhile, our findings here, together with the aforementioned body of evidence in the literature, suggest the hypothesis that some TGF*β*-treated cells could be insufficiently switched to a mesenchymal phenotype, and therefore continue to maintain Golgi and nucleus features similar to those of untreated cells.

A third consideration is that we measured twelve morphological and positional properties of the Golgi and nucleus. Additional features of these organelles are associated with motility, and when measured and included in the analysis, they could reveal Golgi-nucleus relationships that are altered by TGF*β* signaling. These additional properties include stiffness of the nucleus and the degree of compaction of the Golgi [8, 30].

### Future work

The present work shows that multivariate analysis, specifically principal components analysis (PCA), can provide new insights into the roles and relationships among subcellular organelles during cellular processes, such as fibrillar migration. While organelle features are measured at the single-cell level, it remains a challenge to relate these features to cellular states, such as EMT, at the single-cell level. Measuring EMT state and organelle features in the same individual cells would help to address this challenge. Furthermore, multivariate analyses, such as partial least squares regression (PLSR) can be used to relate organelle features to single-cell behaviors, such as migration speed and persistence [31, 32]. In this manner, one can envision that multivariate analysis of data sets acquired by single-cell imaging and image analysis can reveal how cellular biomolecular state (e.g., EMT) impacts physical organization of organelles at the subcellular level and in turn determines cell migration phenotype.
